# Large volume unresectable locally advanced non-small cell lung cancer: acute toxicity and initial outcome results with rapid arc

**DOI:** 10.1186/1748-717X-5-94

**Published:** 2010-10-15

**Authors:** Marta Scorsetti, Pierina Navarria, Pietro Mancosu, Filippo Alongi, Simona Castiglioni, Raffaele Cavina, Luca Cozzi, Antonella Fogliata, Sara Pentimalli, Angelo Tozzi, Armando Santoro

**Affiliations:** 1 Department of Radiation Oncology, IRCCS Istituto Clinico Humanitas, Milano (Rozzano), Italy; 2 Department of Clinical Oncology, IRCCS Istituto Clinico Humanitas, Milano (Rozzano), Italy; 3 Medical Physics Unit, Oncology Institute of Southern Switzerland, Bellinzona, Switzerland

## Abstract

**Background:**

To report acute toxicity, initial outcome results and planning therapeutic parameters in radiation treatment of advanced lung cancer (stage III) with volumetric modulated arcs using RapidArc (RA).

**Methods:**

Twenty-four consecutive patients were treated with RA. All showed locally advanced non-small cell lung cancer with stage IIIA-IIIB and with large volumes (GTV:299 ± 175 cm^3^, PTV:818 ± 206 cm^3^). Dose prescription was 66Gy in 33 fractions to mean PTV. Delivery was performed with two partial arcs with a 6 MV photon beam.

**Results:**

From a dosimetric point of view, RA allowed us to respect most planning objectives on target volumes and organs at risk. In particular: for GTV D_1% _= 105.6 ± 1.7%, D_99% _= 96.7 ± 1.8%, D_5%_-D_95% _= 6.3 ± 1.4%; contra-lateral lung mean dose resulted in 13.7 ± 3.9Gy, for spinal cord D_1% _= 39.5 ± 4.0Gy, for heart V_45Gy _= 9.0 ± 7.0Gy, for esophagus D_1% _= 67.4 ± 2.2Gy. Delivery time was 133 ± 7s. At three months partial remission > 50% was observed in 56% of patients. Acute toxicities at 3 months showed 91% with grade 1 and 9% with grade 2 esophageal toxicity; 18% presented grade 1 and 9% with grade 2 pneumonia; no grade 3 acute toxicity was observed. The short follow-up does not allow assessment of local control and progression free survival.

**Conclusions:**

RA proved to be a safe and advantageous treatment modality for NSCLC with large volumes. Long term observation of patients is needed to assess outcome and late toxicity.

## Background

Lung cancer remains the major cause of cancer-related mortality worldwide. Non-small cell lung cancer (NSCLC) account for at least 80% of all lung tumors and about 30% of them present with unresectable locally advanced disease at diagnosis (stage IIIA-IIIB) [1]. Until the mid 1980s standard treatment of patients with inoperable locally advanced NSCLC consisted of radiotherapy (RT) alone with a median survival time of 10 months[1]. From data about lung cancer population diagnosed in the second half of 1990s, overall survival at one and two years was estimated of 36% and 12% respectively[2].

Rates at 2 and 5 years of 15% and 5% respectively [3]. In attempts to improve the survival in these patients, chemotherapy was added to external beam irradiation. Several trials have been positive in favour of combined therapy [4-6]. More recently, other clinical trials have shown that, in selected patients (good performance status, age ≤ 75 years and minimal weight loss) concomitant platinum-based chemo-radiotherapy is feasible with improvement in progression-free survival and overall survival (OS) in comparison with sequential chemo-radiotherapy (OS 4 years 21% vs. 14%) [7]. However, survival for patients with unresectable locally advanced NSCLC is extremely poor with high rates of loco-regional failure. Recent trials suggest that dose escalation RT could improve loco-regional control with likely benefit on overall survival [8-10]. Rengan et al reviewed the treatment of stage III tumors with large gross tumor volumes (GTV) using 3D-CRT, founding 10 Gy increase in dose to be correlated with a 36.4% decrease in local failure rates [9]. Unfortunately, the large volume of tumor makes dose escalation difficult when using 3D-CRT, since, to avoid treatment related complications such as severe pneumonitis, it is necessary to keep the mean lung dose (MLD) below 20Gy approximately [11].

More recently, the development of intensity modulated radiotherapy (IMRT) makes it possible to deliver a high therapeutic effective dose to the target volume with maximum preservation of surrounding normal tissues. Many studies have compared IMRT and 3D-CRT plans for patients with large unresectable locally advanced NSCLC. Results show statistically significant differences in V20, V30 and MLD for contralateral lung with the values in the IMRT plans being lower. The benefits seemed most pronounced in medium to large tumours [12].

A recent retrospective clinical trial of MSKCC reported a 2-year local control and overall survival of 58% and 58% respectively and a median survival time of 25 months for patients with inoperable stage III lung cancer and treated with 70 Gy (range 60-90 Gy) using IMRT [13]. The results of this study suggest that dose escalation could have an effect not only on local control but also on survival.

To implement dose escalation strategy on NSCLC, advanced IMRT delivery technologies such as Helical Tomotherapy were used and compared with three-dimensional conformal radiotherapy in the clinical practice[14,15]. Both Helical Tomotherapy, and to a lesser extent conventional three-dimensional conformal radiotherapy, have shown the potential to significantly decrease radiation dose to lung and other normal structures in the treatment of NSCLC, providing important implications, in terms of acceptable acute toxicities recorded, for dose escalation strategies in the future[15,16]. In a report of volumetric changes, measured in the primary tumor on megavoltage-computed tomography (MVCT) during chemoradiation, Helical Tomotherapy showed also to be effective in reduce tumour volume in NSCLC[17].

RapidArc(RA), a volumetric modulated arc therapy based on the original investigation of K. Otto [18], has been recently introduced in clinical practice in several institutes after an intensive validation at planning level, compared to IMRT or other approaches, in a series of studies including brain tumours, prostate, head and neck, mesothelioma, cervix uteri and other indications [19-25].

RA is implemented as the Progressive Resolution Optimisation (PRO) algorithm in the Eclipse planning system by Varian Medical System (Palo Alto, California, USA). The optimisation process is based on an iterative inverse planning process that aims to simultaneously optimise the instantaneous multi-leaf collimator (MLC) positions, the dose rate, and the gantry rotation speed in order to achieve the desired dose distribution.

In this study, our aim was to investigate the potential of RA to deliver a therapeutic dose to large volume unresectable NSCLC. We also investigated the possibility of sparing lung tissue, esophagus, heart and spinal cord using RA with the aim of paving the way for further study of dose escalation In the present study, acute toxicities and initial outcome results, were evaluated and reported.

## Methods

### Patients and procedures

This study includes patients with large volume unresectable locally advanced NSCLC (Stage IIIA-IIIB). From May 2009 to September 2009, 24 patients referred to our institution for NSCLC underwent volumetric modulated arc therapy by RA technique. Of these patients 19 were men and 5 women, with a median age of 67 years (range 43-84 years). The total volume of CTV and PTV were recorded for all patients. Specific patients' characteristics are reported in table 1.

**Table 1:** Summary of patients characteristics at treatment start. Values are expressed in number of patients when not otherwise specified

Number of patients		24
**Sex**	**Males**	19
	**Females**	5

**Age [years]** **(median and range)**		67 y (43-84)

**Histology**	**Adenocarcinoma**	12
	**Squamous**	**7**
	**NSCLC NAS**	5
		
**Stage**	**IIIA**	11
	**IIB**	13

**Treatment**	**Sequential**	8
	**Concurrent Chemotherapy**	11
	**RT alone**	5

**Radiation Dose Prescription**	**66 Gy/33fractions**	22
	**60 Gy/30fractions**	1
	**50 Gy/25fractions**	1

The patient's general conditions (age, performance status, weight loss and co-morbidity) were recorded. Total body computed tomography (CT) scan, FDG positron emission tomography (PET) and bone scans were performed in each patient before treatment. In the present population of study, FDG PET was not performed in treatment position and PET images were used by clinician only to obtain a complete stadiation of the patients before treatment. Pathological diagnosis was made by CT-guided fine needle biopsy in 8 patients; 6 patients underwent mediastinoscopy; 4 patients underwent thoracotomy and 6 patients underwent bronchoscopy. Sequential or concomitant chemotherapy was performed in patients of one or more of the following: i) age ≤ 75 years, ii) PS 0-1, iii) minimum weight loss (< 10% 6 months before diagnoses) and iv) Absence of important co-morbidity. Radiotherapy alone was prescribed in patients of one or more of the following:.i) age ≥ 75 years, ii) PS 1-2, iii) minimum weight loss (> 10% 6 months before diagnoses.

All patients received a planning CT scan and were immobilized in a supine position within a personal body-fix pillow. During the scan, and the treatment, patients breathed freely, with the indication to maintain a breathing cycle as regular as possible. The Gross Tumor Volume (GTV) consisted of all known sites of disease with no elective nodal targets. The GTV was defined "large" if it was ≥ 100 cc. The PTV was defined applying an isotropic margin of 8 mm from the primary tumor and of 5 mm from the involved regional lymph nodes. The protocol of treatment started with patients presenting stage III lung cancer in the upper quadrant, and with target volume: > 400 cm^3^. According to Liu et al. [26] the motion of these lesions is lower than 3 mm, therefore the use of 4D techniques is unnecessary. Furthermore daily kV-cone beam CT (CBCT) is performed before RT treatment in order to verify the correct patient position and the target motion (considering the CBCT as a slow CT that includes the lesion motion). Despite daily image guided have already shown in lung the advantage to quantify the volumetric tumour response during treatment [17], in the present study CBCTs were utilized only for patient daily set-up correction.

Organs at risk routinely considered in these patients are contra- and ipsi-lateral lungs, heart, spinal cord and oesophagus. In addition, for all intensity modulated patients, the Healthy Tissue (HT) was defined as the patient's volume included in the CT dataset minus the PTV volume. Volumes are reported in tables 2 and 3.

**Table 2:** Summary of DVH analysis for CTV and PTV

	Objective	RA	
		**CTV** **(mean+1SD)**	**PTV** **(mean+1SD)**

Volume [cm^3^]		299 ± 175	818 ± 206
Mean [%]	100	101.0 ± 1.2	100.0 ± 0.0
D1% [%]	< 107%	105.6 ± 1.7	105.6 ± 1.6
D5-95% [%]	Minimize	6.3 ± 1.4	9.1 ± 1.3
D99% [%]	> 95%	96.7 ± 1.8	91.6 ± 2.2
V95% [%]	100	99.6 ± 0.6	94.6 ± 4.1
V107% [%]	0	0.7 ± 1.3	0.7 ± 1.7

**Table 3:** Summary of DVH analysis for organs at risk

	Objective	RA
		**Ipsi-lateral lung**
**Volume [cm^3^]**	-	1717 ± 549
**Mean [Gy]**	Minimize	30.4 ± 7.0
**V_20Gy _[%]**	Minimize	52.8 ± 8.9
		**Contra-lateral lung**
**Volume [cm^3^]**	-	1945 ± 517
**Mean [Gy]**	< 15 Gy	13.7 ± 3.9
**V_20Gy _[%]**	< 20-30%	21.1 ± 6.1
		**Spinal Cord**
**Volume [cm^3^]**	-	39 ± 16
**D_1% _[Gy]**	< 45 Gy	39.5 ± 4.0
		**Heart**
**Volume [cm^3^]**	-	715 ± 161
**V_45Gy _[%]**	< 30%	9.0 ± 7.0
**V_50Gy _[%]**	< 20%	6.9 ± 6.3
		**Esophagus**
**Volume [cm^3^]**	-	37 ± 13
**V_55Gy _[%]**	< 30%	33.3 ± 10.0
**D_1% _[Gy]**	< 70 Gy	67.4 ± 2.2
		**Healthy tissue**
**Volume [cm^3^]**	-	29588 ± 7788
**Mean [Gy]**	-	9.5 ± 2.7
**V_10Gy _[%]**	-	27.6 ± 7.7

D_x% _= dose received by the x% of the volume; V_x% _= volume receiving at least x% of the prescribed dose; CI = ratio between the patient volume receiving at least 95% of the prescribed dose and the volume of the total PTV. DoseInt = Integral dose, [Gy cm^3 ^10^5^]

All patients were treated with conventional fractionation (2 Gy/day) with no planned treatment breaks. Total dose prescription was 66Gy/33 fractions (with the exception of two patients, one receiving 50Gy and one 60Gy due to OARs limiting factors). In all cases dose normalization was set to mean dose to PTV.

Plans were optimized for two partial isocentric arcs for a Clinac 2100 equipped with a Millennium-120 MLC (120 leaves with a resolution at isocentre of 5 mm for the inner 20 cm and 10 mm for the outer 2 × 10 cm) and a beam energy of 6MV. The arc lengths were set in order to avoid entrance from the contra-lateral lung (depending on target location and extension), and to avoid the posterior entrances, where the couch bars were positioned. For further details on the RapidArc technique see references [15,16].

Plan optimization was performed requiring PTV coverage of 95%-107%. With regard to OARs, the primary objectives were: Spinal cord: D_1% _< 46Gy; Contra lateral lung: V_20Gy _< 30%, mean dose < 15Gy; Oesophagus: D_1% _< 70 Gy. Secondary objectives: Ipsilateral lung: V_20Gy _as low as possible

Oesophagus: V_55Gy _< 30%; Heart: V_50Gy _< 20%, V_45Gy _< 30%.

If the primary objectives were not fulfilled, the prescribed dose was reduced. One patient received 60Gy and another only 50Gy due to organs at risk dose limiting factors.

All dose distributions were computed with the Analytical Anisotropic Algorithm (AAA, version 8.6) implemented in the Eclipse planning system with a calculation grid resolution of 2.5 mm.

### Outcome evaluation

All patients were evaluated weekly with physical and hematologic examination during radiation treatment. Acute and late toxicity events were scored according to the radiation therapy oncology group (RTOG) and the European organization for research and treatment of cancer (EORTC) criteria [27]. Acute reactions included those occurred during or within the first 4 months after the start of radiation treatment and late complications those occurred or persisted after 4 months.

Evaluation of tumor response was performed 45 days after the end of treatment and then every 3 months thereafter with total body CT and/or PET/CT scans. Tumor response was defined according to the Response Evaluation Criteria in Solid Tumor (RECIST) [28]. Clinical and radiographic evidence of local or distant tumor recurrence was recorded.

### Data analysis

Technical features of treatments have been reported in terms of main delivery parameters (number of arcs, control point size, MU, MU/deg and MU/Gy, Dose Rate, Gantry speed, Collimator angle, beam-on and treatment time); beam-on and treatment times are defined without inclusion of patient positioning and imaging procedures and were scored from the record and verify electronic system. Results of pre-treatment plan quality assurance are reported as Gamma Agreement Index (GAI), i.e. the percentage of modulated field area passing the γ-index criteria of Low [29] with thresholds on dose difference set to Δ = 3% of the significant maximum dose, and on Distance to Agreement set to DTA = 3 mm. Measurements and analysis were performed by means of the GLAaS methodology described in [30,31] based on absorbed dose to water from EPID measurements.

Dosimetric quality of the treatments was measured from dose volume histogram (DVH) analysis. For PTV the following data was reported: target coverage (D_1%_, D_99%_, V_95%_, V_107%_), homogeneity (D_5%_-D_95%_). For OARs, the mean dose, the maximum dose (D_1%_) and appropriate values of V_xGy _(volume receiving at least × Gy) were scored.

## Results

Twenty-four patients with large volume NSCLC were treated with a median dose of 66 Gy (range:50-66 Gy) or more using RA from May 2009 to September 2009. All patients had tumors that were unresectable for site and stage. Eleven patients were stage IIIB and 13 patients stage IIIA. Eleven selected patients (age < 70 years, minimum weight loss < 10% 6 months before diagnosis, and PS 0) were treated with concurrent platinum-based chemo-radiation therapy. Eight patients were treated with sequential chemo-radiotherapy, and 5 patients with RT alone because unfit for chemotherapy or elderly.

Only early results are available regarding clinical outcomes. As planned, there were no interruptions during the course of the radiation treatment. No patients had acute skin toxicity. Esophageal toxicity was mild. No Grade 3 toxicity occurred. Grade 1 and Grade 2 acute esophageal toxicity occurred in 22/24 and 2/24 patients respectively. Persistent cough requiring narcotic and antitussive agents occurred in 10/24 patients. For sub-acute toxicity, asymptomatic pneumonia occurred in 6 patients (four grade 1, and two grade 2) who underwent concurrent chemo-radiotherapy at 45 days from the end of treatment and regressed with antibiotic therapy, without hospitalization. Compared with exclusive radiotherapy group, hematologic toxicity was higher in Chemoradiation one, but there was not acute toxicity > Grade 3 or interruptions. There wasn't an important effect of side effects on weight loss for the population of study and PS was not significantly reduced during treatment. The median follow-up time was 6 months.

**Figure 1:**
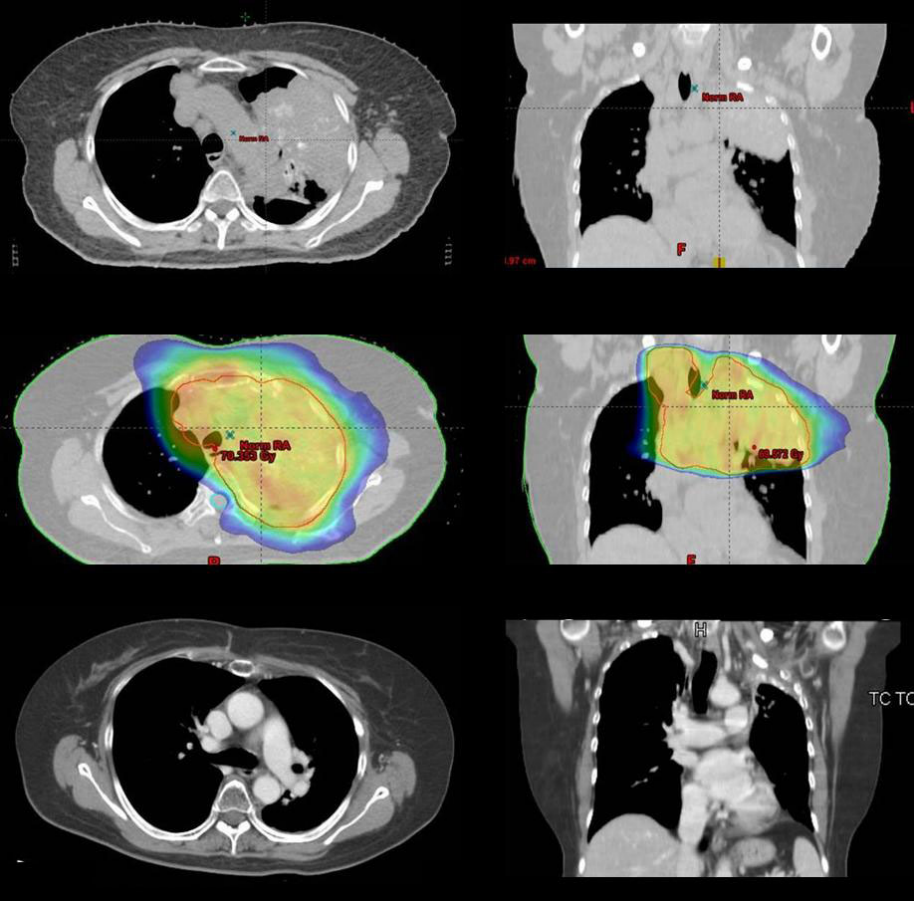
**A case of partial remission**. Up: pre-treatment; Center: dose distribution; Down: 6 months after end of the therapy.

A partial remission > 50% was seen at three months in 56% (14/24) of patients, a partial remission < 50% in 22% (5/24) and stable disease in 22%. No progressive disease occurred. Figure 1 shows a case of significant remission (> 75%) at 5 months after completion of radiotherapy as detected with routing CT scan during follow-up.

**Figure 2:**
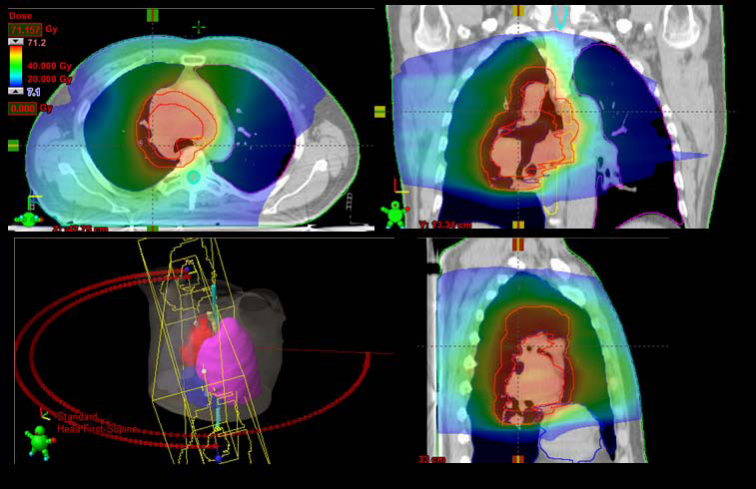
**Isodose distributions for one example patient for an axial plane, sagittal and coronal views**. Doses are shown in colorwash within the interval from 7 to 71Gy. GTV, PTV, and organs at risk are outlined as solid lines. Figure legend text.

**Figure 3:**
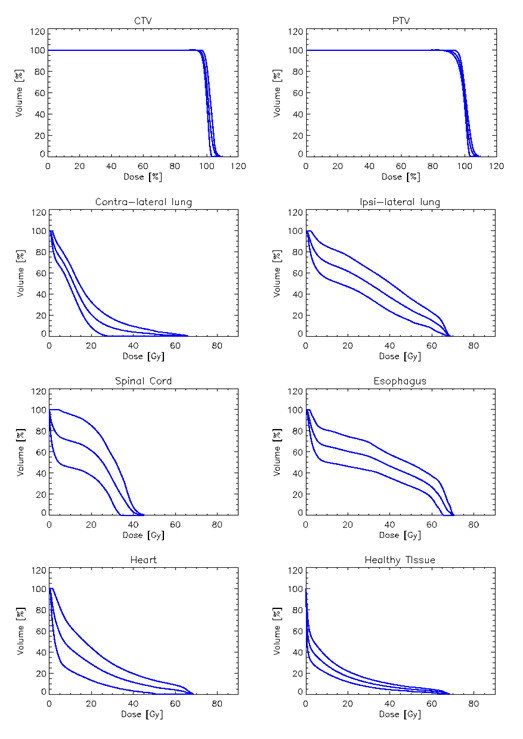
**Average dose volume histograms for GTV, PTV, organs at risk and healthy tissue**. Dashed lines represent inter-patient variability at 1 standard deviation.

Figure 2 shows examples of dose distributions for one patient. Colour wash is in the interval from 7 to 71Gy. GTV, PTV and organs at risk are outlined as solid lines in the images. Figure 3 reports the average dose volume histograms for GTV, PTV, organs at risk and healthy tissue. Lines represent the inter-patient variability at one standard deviation.

Table 4 summarizes the technical features of the treatment characteristics. Results of the DVH analysis are reported in Table 2 for PTV and GTV, and in table 3 for organs at risk and healthy tissue, together with the specific objectives.

**Table 4:** Technical characteristics of RapidArc and conventional plans

	RA
**Number of arcs or fields**	2
**Arcs length [°] *whole plan***	401 ± 166
**Beam energy**	6 MV
**Delivery time [s]**	133 ± 7
**MU/fraction**	391 ± 68
**MU/Gy**	190 ± 38
**Dose Rate [MU/min]**	239 ± 51
**Gantry speed [deg/sec]**	4.79 ± 0.02
**Collimator angle [°]**	19 ± 8
**Mean leaf aperture [cm]**	5.3 ± 1.5
**Mean CP area [cm^2^]**	98 ± 30
**Mean field area [cm^2^]**	337 ± 91

MU: monitor units, CP: control point

Dosimetric data showed that RapidArc obtained the achievement with respect to planning objectives for most of the parameters considered. In particular, the target coverage and dose homogeneity are well achieved, even in regions with highly demanding heterogeneities (GTV well within the thresholds of 95% and 107%). In terms of organs at risk the contralateral lung presented promising results, while keeping the maximum significant dose to the spinal cord well below the tolerance level. Heart irradiation was not of major concern for the selected patients due to the relatively cranial target location. The oesophagus volume irradiated to doses higher than 55Gy is in average higher than objective by about 3%, but this value is largely dependent on the organ volume included inside the target volume. As expected from an intensity modulation arc technique the low dose bath is not negligible, showing about 8000 cm^3 ^of tissue irradiated at 10Gy dose level.

Pre-treatment quality assurances of RA plans resulted in an average gamma agreement index GAI 3% superior to the acceptance threshold of 95% set as a reference in our institute.

## Discussion

Despite an improvement in survival the prognosis of patients with unresectable local advanced NSCLC (stage IIIA-IIIB) remains poor both for locoregional failure and for distant metastatic disease. Concurrent chemo-radiation is at present the standard of care. Given that local recurrence is the leading cause of death in this patient population, techniques for improving local control may have a positive impact on survival rates and quality of life. The value of dose escalation was demonstrated in a study from MSKCC; Rengan et al founded that a 10 Gy increased in dose correlated to a 36.4% decrease in local failure rates [9]. Unfortunately, the extension of disease and the presence of surrounding HT makes it often difficult to deliver high doses with curative intent.

Based on the results of an intensive program of pre-clinical investigations performed at planning level [19-25] for assessing the reliability and potential benefit of RA, this technique has been used in clinical practice for a variety of indications at our Institute since November 2008. The present study reports the early findings from the treatment of a group of 24 patients affected by advanced lung cancer irradiated with RapidArc.

The main objective in the initial clinical introduction of RA is the evaluation of the possibility to provide RT treatments respecting a set of planning objectives. These results should be achieved without introducing elements of potential confusion like alterations of the fractionation schemes (acceleration or hypo-fractionation for example) or dose escalation [14]. Further studies will assess the elements of improvement once the safety of the new approach is consolidated in routine practice. These results could enable the activation of a second phase dose trial aiming to push RapidArc towards improved sparing of organs at risk, particularly the contra-lateral lung.

Having achieved the result of respecting or improving most of the planning objectives, RapidArc confirmed also some advantages at a logistical level. It provided a significant efficiency in dose delivery treatment time,. This is a particularly important point for patients presenting advanced lung cancer, who might have breathing difficulties in supine position, or even coughing if lying for a long time: shortening the time spent on the couch permits the treatment for this class of patients.

From the clinical point of view, the data presented here are encouraging, confirming that RA can be considered as a safe modality for this category of patients having proved limited impact in terms of acute toxicity [32,33]. The smoother process of RA and its potential reduction in acute toxicity could also lead to a more uniform duration of treatments, reducing the risk of unscheduled and undue interruptions.

In particular, the authors point out the extension of disease to be greater than other population finding in literature. Sura et al. [13] report mean PTV volume of 459 cm^3^, much smaller than our series, where mean PTV volume was 818 cm^3^, thus corroborating our results.

It is obvious that the present study cannot be considered as conclusive and that long-term observation of patients is needed to define outcome and late toxicity. These preliminary results are encouraging additional experience in this field. Further investigations will aim to look at the long term clinical outcome and late toxicity as well as improvements in sparing of the organs at risk.

## Conclusions

Twenty-four patients with large volume unresectable locally advanced lung cancer were treated with RA at Istituto Clinico Humanitas. Quality of treatments resulted in a general fulfilment of the planning objectives. Clinical outcome for early acute toxicity showed limited events. Future investigations will aim to increase sparing of organs at risk and to look to long-term outcome based on the fact that the first phase has achieved the primary goal of demonstrating the safety and efficacy of RA.

## Competing interests

Dr. L. Cozzi is Scientific Advisor to Varian Medical Systems and is Head of Research and Technological Development to Oncology Institute of Southern Switzerland, IOSI, Bellinzona.

Other authors do not have conflict of interest to declare.

## Authors' contributions

MS, PM, LC and AF coordinated the entire study. Patient accrual and clinical data collection was done by FA, SC, RC, SP, AR, SP, AS. Data analysis, physics data and treatment planning data collection was conducted by PN, PM, SP, AC, GN, EV and AF. The manuscript was prepared by MS, PN, LC. All authors read and approved the final manuscript.
